# Carbonaceous particle exposure triggered accumulation of Osteopontin/SPP1+ macrophages contributes to emphysema development

**DOI:** 10.1002/mco2.70061

**Published:** 2025-01-28

**Authors:** Lianyong Han, Verena Haefner, Ali Önder Yildirim, Heiko Adler, Tobias Stoeger

**Affiliations:** ^1^ Institute of Lung Health and Immunity (LHI) Comprehensive Pneumology Center (CPC) Helmholtz Zentrum München, German Research Center for Environmental Health, Neuherberg, Germany Member of the German Center of Lung Research (DZL) Munich Germany; ^2^ Institute of Experimental Pneumology (IEP) Medical Faculty of Ludwig Maximilians University Munich Munich Germany; ^3^ Institute of Asthma and Allergy Prevention, Helmholtz Zentrum München, German Research & Center for Environmental Health, Neuherberg, Germany; Walther‐Straub‐Institute of Pharmacology and Toxicology & Faculty of Medicine Ludwig‐Maximilians‐University Munich, Member of the German Center of Lung Research (DZL) Munich Germany

1

Dear Editor,

Chronic obstructive pulmonary disease (COPD) is an inflammatory disease characterized by airway obstruction and loss of alveolar surface, together resulting in progressive and irreversible airflow limitations and shortage of breath. Chronic bronchitis and emphysema are two major phenotypes of the disease. Cigarette smoke (CS) is a long‐known cause of COPD, and it accounts for more than 70% of COPD cases, as reported by WHO,^1^ but the contribution of indoor and outdoor air pollution is increasingly acknowledged.^2^ As one of the major air pollutants, ambient particle inhalation has been widely reported to contribute to several chronic lung diseases, in particular COPD. Typically, animal studies revealed that the inhalation of soot‐like carbonaceous nanoparticles (CNPs) can cause both local inflammation in the lung as well as systemic inflammation, allowing the application of CNP as a representative environmental and combustion‐derived particle to investigate its potential toxicological effects as well as the association with chronic lung diseases. Our previous study showed that repeated exposure of mice to CNP, mimicking a human urban exposure scenario under the condition of latent gammaherpesvirus infection (particles as a second hit on top of latent virus infection), caused interstitial inflammation, alveolar injury and cell death leading to progressive alveolar air space enlargement, thereby demonstrating a crucial contribution of environmental particle exposure to lung emphysema development.^3^, ^4^ Several mechanisms have been suggested to contribute to epithelial cell damage by air pollutants and inhaled particles.

Secreted phosphoprotein 1 (SPP1), also known as Osteopontin (OPN), is a matricellular protein expressed by many cell types, exhibiting an important role in various inflammatory responses. For instance, it has been shown that inhalation of CNP or CS caused OPN release in the airways of a murine COPD model.^5^ Furthermore, in many observational studies, OPN release is also increased in various body fluids including sputum and plasma from COPD patients compared to healthy individuals.^6^, ^7^ While plasma OPN levels have been associated with exposure to air pollution, which in turn was linked to worsening emphysema, the mechanistic link between particle exposure triggered SPP1 and COPD development has so far only been drawn for CS.

To study the expression of SPP1 during COPD development, we analyzed *SPP1* messenger RNA (mRNA) levels from either emphysema or COPD patients across four different cohorts. Consistently, we found that *SPP1* is significantly elevated in lung tissue of either emphysema or COPD patients compared to controls (Figure 1A), supporting the clinical relevance of SPP1 induction during COPD development. Experimentally, in a CS‐induced mouse COPD model, *Spp1* was also found highly induced in the lung, and as shown by the single cell transcriptomics (Figure 1B), expression predominantly maps to CD11b (*Itgam*) positive lung macrophages, and only slightly to CD11c (*Itgax*) positive cells. Supported by immunohistological examinations, monocyte‐derived alveolar macrophages seem a major source of pulmonary SPP1 levels.^8^ Indeed, *Spp1* is persistently upregulated in those lung macrophages upon long‐term CS exposure for up to 6 months (Figure 1B). Taken together, SPP1 is highly induced in both COPD patients and the COPD mouse model and mainly localizes to the macrophages in the lung.

**FIGURE 1:**
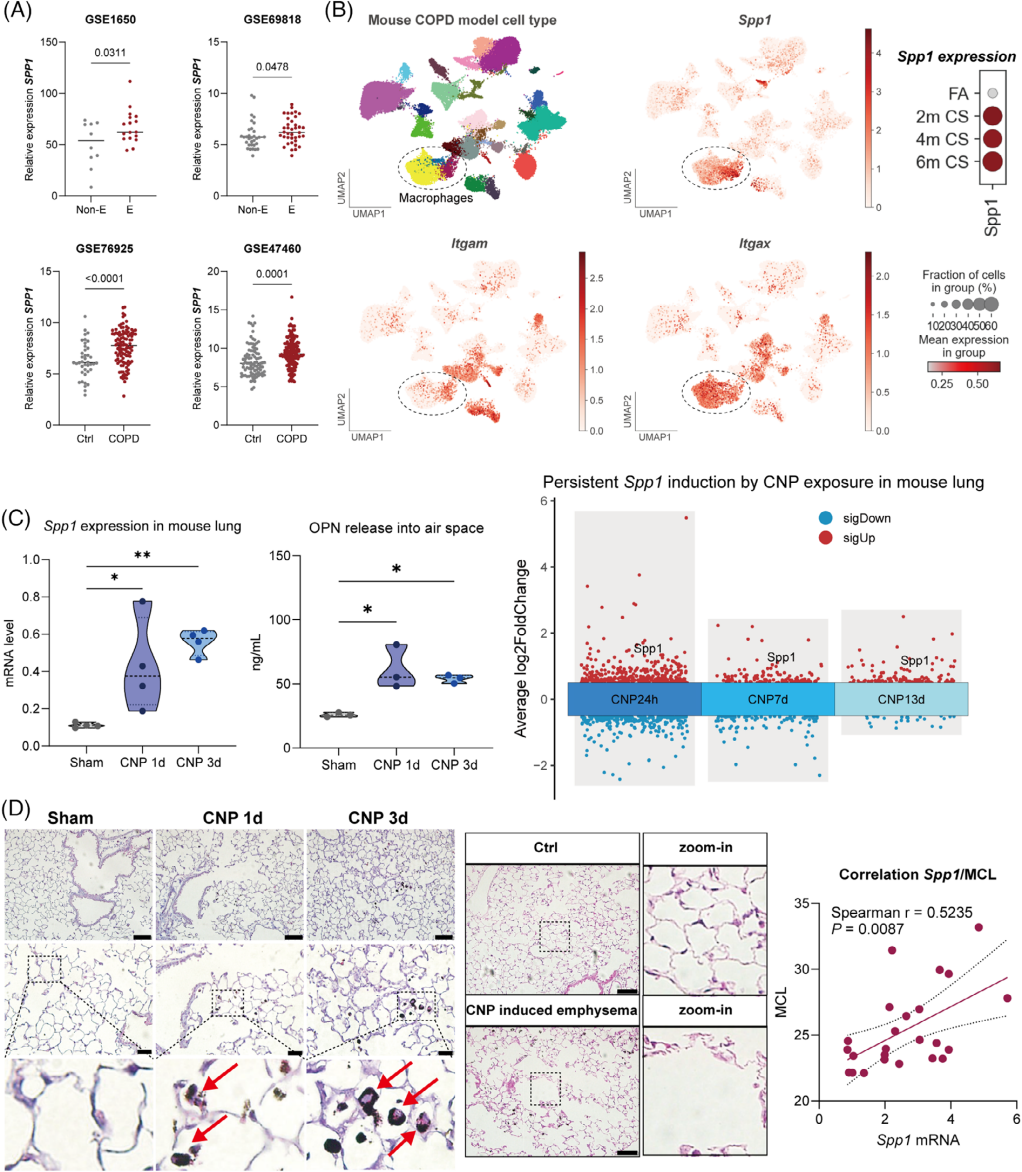
Carbonaceous particle exposure triggered accumulation of Secreted phosphoprotein 1+ (SPP1+) macrophages contributes to emphysema development. (A) The expression of *SPP1* in the lungs of non‐emphysema and emphysema (upper two charts; Non‐E: non‐emphysema, E: emphysema) as well as healthy individuals (Ctrl) and chronic obstructive pulmonary disease (COPD) patients (lower two charts). Each dot represents an individual case, and the relative expression levels are shown. Data are taken from GSE1650 (upper left chart; *n* = 10 non‐emphysema and *n* = 17 emphysema patients), GSE69818 (upper right chart; *n* = 32 non‐emphysema and *n* = 38 emphysema patients), GSE76925 (lower left chart; *n* = 40 healthy and *n*  =  111 COPD patients) and GSE47460 (lower right chart; *n* = 91 healthy and *n* = 145 COPD patients). Values are shown as mean ± SEM. Data were analyzed with either a student's *t*‐test or a non‐parametric test between two groups. *p*‐Values are shown on each plot. (B) The localization of *Spp1, Itgam* (CD11b), and I*tgax* (CD11c) visualized by UMAP in a cigarette smoke (CS)‐induced COPD model (GSE185006; left and middle panel). Mice were exposed to filtered air (FA) or CS for 2, 4, and 6 months, lung tissue was harvested to perform single‐cell transcriptomics. The persistent induction of *Spp1* by CS exposure after 2, 4, and 6 months in the macrophage population is shown by dotplot (right panel) The dot size represents the fraction of cells in the treatment group, the color represents the mean expression of the gene in the treatment group. (C) The expression of *Spp1* messenger RNA (mRNA) as measured by quantitative polymerase chain reaction (qPCR) in mouse lungs exposed to carbonaceous nanoparticle (CNP) for 1 and 3 d (left panel). The release of SPP1 (OPN) protein into alveolar air space as measured by enzyme‐linked immunosorbent assay (ELISA) from BAL fluid samples of mice after exposure to CNP for 1 and 3 d (middle panel). Values are shown as means ± SEM (*n* = 3 for qPCR and *n* = 4 for ELISA). One‐way analysis of variance (ANOVA) followed by Tukey's multiple comparisons test was used for statistical analysis. *p < 0.05. **p < 0.01. The microarray data of the persistent upregulation of *Spp1* in mouse lungs after exposure to CNP for 1, 7, and 13 d shown in the right panel. Gene expression is visualized by a multi‐volcano plot. Red dots represent significantly upregulated genes, and blue dots represent significantly downregulated genes. (D) The accumulation of SPP1+ macrophages in the mouse lung after exposure to CNP at d1 and d3. Immunohistochemistry (IHC) staining identifies the SPP1 localization. Scale bar: 50 µm in 20x magnification images and 20 µm in 40x magnification images. In a repeated CNP exposure mouse model, CNP caused emphysema‐like changes in the lung (middle panel). Scale bar: 50 µm. The correlation between *Spp1* expression level and mean chord length (MCL) (right panel). The correlation analysis was performed using the Spearman correlation method, Spearman's *r*‐value and *p*‐value were shown.

As shown in our previous virus‐particle second‐hit study, CNP exposure caused interstitial inflammation, alveolar injury, and cell death as well as progressive emphysema‐like changes.^4^ We thus investigated whether *Spp1* could be also induced by CNP and thereby contribute to environmental particle exposure related COPD development. After intratracheal exposure of mice to 50 µg CNP, we found that pulmonary *Spp1* mRNA was progressively induced from 1 d to 3 d (Figure 1C, left panel). Airspace concentrations of OPN protein in bronchoalveolar lavage (BAL) fluid, also remained elevated till 3 d (Figure 1C, middle panel), the time point where the neutrophilic inflammation was to a great extent already resolved. In fact, *Spp1* lung levels even remained elevated till two weeks after CNP exposure (Figure 1C, right panel), as shown in the volcano plot. In the murine COPD model (Figure 1B), CS exposure triggered *Spp1* expression localizes predominantly to macrophages. Similarly, SPP1 immunohistochemistry (IHC) staining on CNP exposure approved SPP1 induction within 1 d until 3 d in particle‐laden alveolar space macrophages (Figure 1D, left panel, red arrows). We have previously described in a 2nd hit model, that repeated CNP exposure of gammaherpesvirus‐infected mice caused lung emphysema as quantified by air space Mean Chord Length (MCL). Representative images from H&E staining are depicted in Figure 1D (middle panel). Interestingly lung *Spp1* mRNA levels correlate with emphysema‐like changes, that is, the MCL value (*p* = 0.0087), indicating the strong association between SPP1 induction caused by CNP exposure and emphysema development (Figure 1D, right panel). Mechanistically, Shan and colleagues have elegantly shown in a CS‐induced mouse emphysema model, that by lung phagocytes released SPP1 mediates emphysema development via activating Th17 signaling, as Th17 cells and emphysema development was reduced in OPN deficient mice.^9^ Also, the deactivation of PPARγ, a negative regulator of Th17 differentiation and *Spp1* expression, leads to lung inflammation and emphysema development. In this context, the authors suggested that carbon black, a particulate constituent of CS, might cause emphysema through the proposed Th17 pathway.^10^ Together these findings highlighted the impact of SPP1 on lung emphysema development related to CS and carbonaceous particle pollution and uncovered its potential molecular mechanism.

Considering that over 90% of the global population is still living in areas where air pollution levels exceed WHO guidelines of 10 µg/m^3^ for PM2.5, further aggravated by occupational exposure, the persistent particle challenges in the real‐world pose long‐term damage and injury to the lung and also alter the immunoregulation machinery of our body, which finally contributes to the development of chronic lung diseases. In the present study, we highlight the contribution of carbonaceous particle exposure to emphysema and COPD development. Furthermore, the presented data demonstrated that *SPP1* expression is increased in the lungs of COPD patients as well as in the CS‐induced COPD mouse model and localizes to lung macrophages. In an environmental particle exposure scenario, soot‐like CNP particles not only induce *Spp1* expression in the lung but also release OPN into the alveolar air space, thereby potentially participating in lung inflammation and injury. SPP1+ macrophages localize to the alveolar space and interstitium, naturally in proximity to the fragile alveolar wall, and obviously contain a high burden of particle agglomerates, similar to the CNP and CS exposure model. Noteworthy, carbonaceous particle exposure‐induced *Spp1* expression in the animal model positively correlated with emphysema‐like changes, highlighting the contribution of SPP1 to the development of air pollution‐related emphysema.

In summary, our data suggests a crucial contribution of SPP1+ macrophages to environmental particle pollution‐related emphysema development and thus SPP1+ macrophages may provide a potential preventive target for COPD.

## AUTHOR CONTRIBUTIONS

**Lianyong Han**: designed the experiment and analyzed all the data. **Verena Haefner**: performed data analysis. **Lianyong Han**: drafted the manuscript. **Ali Önder Yildirim, Heiko Adler and Tobias Stoeger**: revised the manuscript. All authors have read and approved the final manuscript.

## CONFLICT OF INTEREST STATEMENT

The authors declare no conflict of interest.

## ETHICS STATEMENT

All animal experiments in the study were in compliance with protocols approved by the local Animal Care and Use Committee (District Government of Upper Bavaria; permit numbers: 124/08 and 67/2015).

## Supporting information




Supporting Information

## Data Availability

All the public human patient data included in the study can be accessed via GEO at NCBI (GSE1650, GSE69818, GSE76925, and GSE47460). The mouse COPD single‐cell RNA sequencing data is accessible via GEO (GSE185006). Microarray data on mouse lungs exposed to CNP were deposited in GEO in NCBI (GSE79501 and GSE223818).
