# Supplementary material for: Carbonaceous particle exposure triggered accumulation of Osteopontin/SPP1+ macrophages contributes to emphysema development
Source: MedComm (2020). 2025 Jan 28;6(2):e70061. doi: 10.1002/mco2.70061 (PMC11774239; doi:10.1002/mco2.70061)
Supplement: Supplementary file 1 — Supporting Information [file MCO2-6-e70061-s001.docx]

**Supplementary information**

**Carbonaceous particle exposure triggered accumulation of Osteopontin/SPP1+ macrophages contributes to emphysema development**

*Lianyong Han, Verena Haefner, Ali Önder Yildirim, Heiko Adler, Tobias Stoeger*

**Methods**

***Nanoparticles and materials***

Carbonaceous spherical nanoparticles (CNP; Printex90, Degussa, Frankfurt, Germany) was used in this study. CNP was dispersed in ultrapure water to reach a stock solution at 1 mg/ml. The further dispersion was performed by 5 min in ice-cold water bath as well as 30 s at probe sonication. For *in vivo* experiments in the study, mice were instilled with 50 μg of CNP.

***Animal experiments***

Female C57BL/6 mice were purchased from Charles River Laboratories (Sulzfeld, Germany) and housed in individually ventilated cages during the experiments. Mice were instilled with 50 μg of CNP or an equal amount sterile H_2_O as sham control per mouse as described previously ^1,2^. For the acute exposure experiments, samples were collected after 1 and 3 d, and for chronic exposure experiments, sample harvest was performed after 1, 7 and 13 d. In general, lung tissues were harvested for either RNA or protein isolation to measure target gene or protein, isolated RNA was also used to perform microarray. ~~or histological analysis~~ Lung tissue was also fixed with 4% paraformaldehyde (PFA) solution for histological analysis. Bronchoalveolar lavage (BAL) fluid was also collected to measure OPN (SPP1) protein release. All animal experiments were in compliance with protocols approved by the local Animal Care and Use Committee (District Government of Upper Bavaria; permit number: 124/08, 67/2015 and 55.2-2532.Vet_02-15-67).

***Lung sections for histological analysis***

Mouse lung tissue was fixed in 4% PFA solution at 4 °C and washed with PBS two times. Then, lung tissue was embedded in paraffin and gets a block. 3 μm slices were cut for further histological staining.

***Immunohistochemistry (IHC)***

Deparaffinization and rehydration to the slices were first done following by the peroxidase blocking, epitope retrieval, blocking as well as antibody incubation steps. The detailed method were described elsewhere before ^1^. Vulcan Fast Red Chromogen Kit (Biocare Medical/Zytomed Systems, Berlin, Germany) was used in the experiment. Slices were later counterstained with Haematoxylin followed by Xylene incubation before mounting. Mouse OPN / SPP1 antibody (Assay designs; Rabbit polyclonal, 1:50 dilution) was used in the study. Representative images were taken with a light microscope (Olympus BX51) including low (20x) and high (40x) magnifications.

***SPP1 protein level measurement by ELISA***

OPN / SPP1 protein level in BAL fluid was measured with Mouse Osteopontin DuoSet ELISA kit (R&D Systems, Inc. Minneapolis, MN) following the instructions of the manufacturer.

***Bulk transcriptomic analysis in mouse lung with Microarray***

Mouse whole lung RNA with QIAGEN RNeasy Mini Kit (QIAGEN GmbH, Düsseldorf, Germany) following the instructions of the manufacturer. RNA concentration was measured with Nanodrop (Thermo Fisher Scientific, Waltham, MA, USA). The Agilent 2100 Bioanalyzer was used to assess RNA quality and only high-quality RNA (RIN > 7) was used for microarray analysis. Total RNA was amplified using the Illumina TotalPrep RNA Amplification kit (Ambion, Life Technologies, Carlsbad, CA, USA) or the WT PLUS Reagent Kit (Thermo Fisher Scientific Inc., Waltham, MA, USA). Amplified cRNA was hybridized to Mouse Ref-8 v2.0 Expression BeadChips (Illumina, San Diego, CA, USA) for the chronic exposure experiment and Mouse Clariom S arrays (Thermo Fisher Scientific, Waltham, MA, USA) for the acute exposure experiment, respectively. Staining and scanning (GeneChip Scanner 3000 7G) was done according to manufacturer’s instructions. For the chronic exposure experiment, data was processed using the GenomeStudioV2010.1 software using the following annotation file (MouseRef-8_V2_0_R3_11278551_A.bgx). For acute exposure experiment, annotated normalized SST-RMA gene-level data was obtained by the Transcriptome Analysis Console (TAC; version 4.0.1.36; Thermo Fisher Scientific, Waltham, MA, USA). Statistical analyses were performed by utilizing the statistical programming environment R (v4.0.4). Genewise testing for differential expression was done employing the limma *t*-test and regulated gene sets were defined by raw p-value < 0.05. To reduce background, gene sets were filtered using DABG p-value < 0.05 in more than half of the samples in at least one group per comparison. Dotplot, barplot and treeplot were used to visualize enriched terms according to the use of ggplot2 package. The chronic exposure experiment array data has been submitted to GEO database at NCBI (GSE79501); acute exposure experiment array data has been submitted to the GEO database at NCBI (GSE223818). The visualization of gene expression by volcano plot from array data was performed with R using ggplot2 package, scRNAtoolVis package.

**Determination of gene expression by qPCR**

Whole cell RNA was isolated with QIAGEN RNeasy Mini Kit (QIAGEN GmbH, Düsseldorf, Germany) following the instructions of the manufacturer. RNA concentration was measured with Nanodrop (Thermo Fisher Scientific, Waltham, MA, USA). Subsequently, RNA was reverse-transcribed using superscript kit (Invitrogen, Waltham, MA, USA). Then, cDNA was used to analyze the target gene expression by real time quantitative PCR using SYBR Green PCR master mix (Thermo Fisher Scientific, Waltham, MA, USA). The determination of mouse *Spp1* gene expression determination was using primer pairs: forward: agctcagaggagaagctt and reverse: cttcagaggacacagcat. *Hprt* was used as a housekeeping gene (forward: gttggatacaggccagactttgt; reverse: cacaggactagaacacctgc). The relative expression level is shown as 2^-ΔCt^, where ΔCt = Ct _target gene_-Ct _housekeeping gene_.

***Single cell RNA sequencing data***

The single cell RNA sequencing data from cigarette smoke (CS) induced COPD mouse model was downloaded via GEO accession: GSE185006). The treatments to the mice contain filtered air (FA) or cigarette smoking for 2, 4 and 6 months. Data analysis was performed using Scanpy package (version 1.8.0), and plotting functions were used to visualize gene expression, by either Uniform Manifold Approximation and Projection (UMAP) or dotplot.

***Human COPD microarray data***

The emphysema or COPD patients’ data were taken from 4 different clinical studies via the GEO accession: GSE1650, GSE69818, GSE76925 and GSE47460. According to the raw data file in NCBI, the expression level of SPP1 in emphysema or COPD lung samples were taken and the statistical analysis was then performed using GraphPad Prism software v10.1.2 (GraphPad Software, Inc., San Diego, CA, USA).

***Statistical analysis***

All values are shown as mean ± SEM. Comparisons between two groups were performed using Student’s *t* test or by non-parameter Mann-Whitney test depending on the data distribution (Data shown in **Figure 1A**). And comparisons between multiplex groups were performed using one-way ANOVA followed by Tukey’s multiple comparisons method (Data shown in **Figure 1C**). Spearman correlation analysis was performed to investigate the correlation between Spp1 mRNA level in mouse lungs and MCL value. All analysis was performed using GraphPad Prism software v10.1.2 (GraphPad Software, Inc., San Diego, CA, USA). *P* value or “*” was shown. * *P* < 0.05, ** *P* < 0.01.

**Reference**

1. Han L, Haefner V, Yu Y, et al. Nanoparticle-Exposure-Triggered Virus Reactivation Induces Lung Emphysema in Mice. *ACS Nano*. Nov 14 2023;17(21):21056-21072.

2. Sattler C, Moritz F, Chen S, et al. Nanoparticle exposure reactivates latent herpesvirus and restores a signature of acute infection. *Part Fibre Toxicol*. Jan 10 2017;14(1):2.
